# The *Helicobacter cinaedi* antigen CAIP participates in atherosclerotic inflammation by promoting the differentiation of macrophages in foam cells

**DOI:** 10.1038/srep40515

**Published:** 2017-01-11

**Authors:** Mario Milco D’Elios, Francesca Vallese, Nagaja Capitani, Marisa Benagiano, Maria Lina Bernardini, Mirko Rossi, Gian Paolo Rossi, Mauro Ferrari, Cosima Tatiana Baldari, Giuseppe Zanotti, Marina de Bernard, Gaia Codolo

**Affiliations:** 1 Department of Experimental and Clinical Medicine, University of Florence, Florence, Italy; 2 Department of Biomedical Sciences, University of Padua, Padua, Italy; 3 Department of Life Sciences, University of Siena, Siena, Italy; 4 Department of Biology and Biotechnology, “C. Darwin”, Sapienza University of Rome, Rome, Italy; 5 Institute Pasteur Italy - Fondazione Cenci Bolognetti, Rome, Italy; 6 Department of Food Hygiene and Environmental Health, University of Helsinki, Helsinki, Finland; 7 Internal Medicine, Department of Medicine-DIMED, University of Padua, Italy; 8 Vascular Surgery Unit, Cisanello University Hospital AOUP, Pisa, Italy; 9 Department of Biology, University of Padua, Padua, Italy

## Abstract

Recent studies have shown that certain specific microbial infections participate in atherosclerosis by inducing inflammation and immune reactions, but how the pathogens implicated in this pathology trigger the host responses remains unknown. In this study we show that *Helicobacter cinaedi* (Hc) is a human pathogen linked to atherosclerosis development since at least 27% of sera from atherosclerotic patients specifically recognize a protein of the Hc proteome, that we named Cinaedi Atherosclerosis Inflammatory Protein (CAIP) (n = 71). CAIP appears to be implicated in this pathology because atheromatous plaques isolated from atherosclerotic patients are enriched in CAIP-specific T cells (10%) which, in turn, we show to drive a Th1 inflammation, an immunopathological response typically associated to atherosclerosis. Recombinant CAIP promotes the differentiation and maintenance of the pro-inflammatory profile of human macrophages and triggers the formation of foam cells, which are a hallmark of atherosclerosis. This study identifies CAIP as a relevant factor in atherosclerosis inflammation linked to Hc infection and suggests that preventing and eradicating Hc infection could reduce the incidence of atherosclerosis.

Atherosclerosis through its complications, namely heart attacks, stroke and renal and limb ischemia, is the major cause of death and early disability in western countries^1^.

The disease is a multistep chronic inflammatory disorder that starts with the accumulation of lipids inside the intima of the medium and large arteries^2^,^3^,^4^. The precise cause of atherosclerosis is not known; however, the evidence that certain genetic traits (familiar hypercholesterolemia), conditions (insulin resistance, hypertension), or habits (smoking) may raise the risk for the disease^5^,^6^, indicates that atherosclerosis is a multifactorial disease.

The hypothesis that pathogens causing chronic infections might promote atherosclerosis and coronary artery disease has received great attention in recent years. In particular, the detection of infectious agents *Chlamydia pneumoniae*^7^,^8^,^9^, *Porphyromonas gingivalis*^10^,^11^, and cytomegalovirus^12^, within human atherosclerotic tissues has raised interest in the role of pathogens favouring the clinical manifestations of atherosclerosis. It is likely that the contribution of microbes relies on the inflammation they elicit as well as on their impact on the adaptive immune response. The *C. pneumoniae* phospholipase D, for example, is able to drive the expression of IL-23, IL-6, IL-1β, TGF-β, and CCL-20 by monocytes, and to activate a Th17 immune response that, together with the Th1, constitutes the main inflammatory signature in human atherosclerotic plaques^9^.

In the last few years, another microbe has drawn the attention for its potential contribution to the development of atherosclerotic lesions, i.e. the bacterium *Helicobacter cinaedi* (Hc). Hc is the most frequently reported non-gastric *Helicobacter* species^13^,^14^,^15^,^16^. Although Hc has been isolated from patients suffering from enteritis and bacteremia, in most cases patients infected by Hc do not display any symptoms other than fever^17^. This observation, together with the difficulty in detecting Hc by conventional culture methods, makes it virtually impossible to determine the exact spread of this bacterium in the general human population^18^,^19^. With respect to the other microorganisms that may have a role in the atherosclerotic process, Hc displays a strong ability to invade the vascular system as a distinctive feature. The bacterium efficiently translocates from the intestinal tract, the primary site of infection, to the vascular system, where it can cause endovascular infections^20^. Cases of endocarditis^21^, myopericarditis^22^ and aneurysms^23^,^24^ have been attributed to Hc infection, and Hc antigens have been found inside macrophages in both mature and immature human atheromas^25^. The etiological role of Hc has been further substantiated by the finding that Hc infection worsens the atherosclerotic lesions in hyperlipidaemic mice^26^. The Hc ability of promoting the accumulation of pro-inflammatory cytokines within the lesions, as well as the activation of macrophages and their differentiation in foam cells^26^, probably play a pivotal role in aggravating plaque formation. The hypothesis that a Hc-specific immune response might also contribute cannot be ruled out as it has not been investigated yet.

Several virulence factors are predicted to be produced by the bacterium, based on the complete genome sequence of Hc^27^. Noteworthy, a pathogenic role in enteritis has been demonstrated for one of them, the cytolethal distending toxin (CDT)^28^, but it is unknown if it may also contribute to the pathogenesis of atherosclerosis.

DNA-binding proteins from starved cells (Dps) are a group of bacterial miniferritins with a nearly spherical dodecameric structure^29^,^30^. The neutrophil activating protein of *Helicobacter pylori* (HP-NAP) belonging to the Dps-like family has a crucial role in the *H. pylori*-gastric chronic diseases as a result of its specific effects on components of the innate and adaptive immune response^31^,^32^. Hc also possesses a *napA* gene predicted to encode a protein highly homologue to HP-NAP^27^,^33^.

We herein identified the product of the Hc *nap*A gene that we named Cinaedi Atherosclerosis Inflammatory Protein (CAIP), a 200 kDa major antigen of the immune response of Hc-infected individuals with atherosclerosis. Considering that atherosclerotic lesions contain large numbers of immune cells, we investigated the role of CAIP in modulating the functional profile of macrophages and T cells, which not only are the most abundant components in the lesions, but also have an established role in the initiation, progression, and development of complications of atherosclerotic plaques^34^.

## Results

### CAIP is an antigen released by *H. cinaedi,* recognized by circulating antibodies of atherosclerotic patients

**Figure 1:**
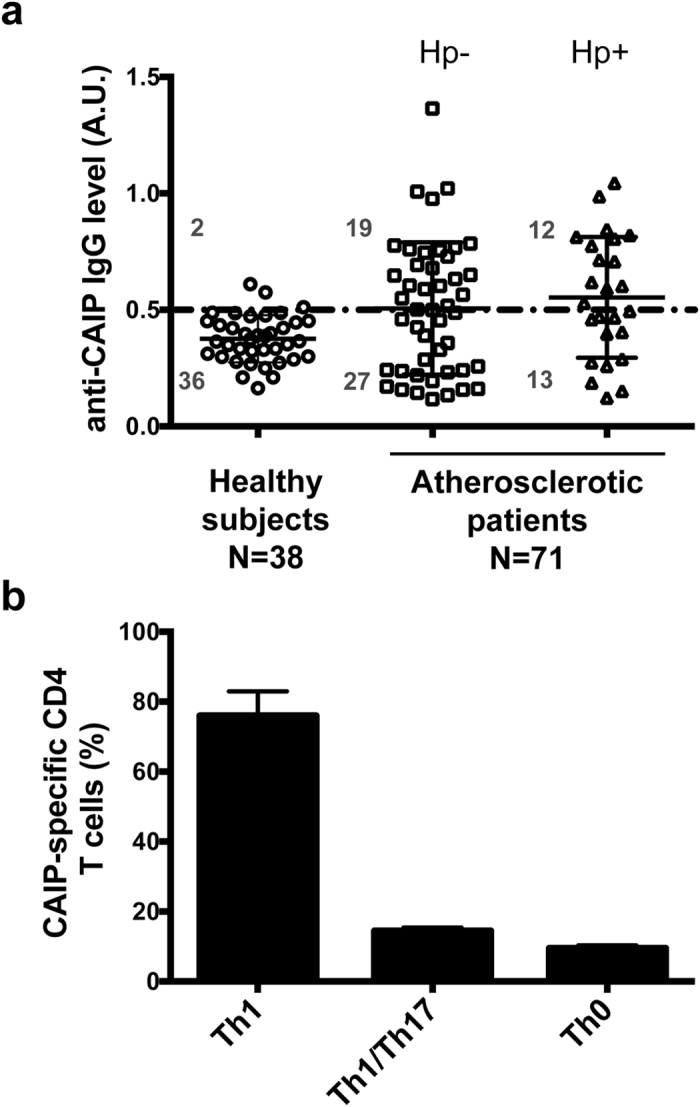
Atherosclerotic patients have both antibodies in the serum and Th1 cells in the plaques that are specific for CAIP. (**a**) Seroconversion against CAIP of 38 healthy subjects and 71 subjects with atherosclerosis is shown. Individuals positive for *H. pylori* infection (Hp+) are distinguished from those that are *H. pylori*-negative (Hp−). ELISA results were considered positive at >5 S.D. above the mean value in healthy subjects, and 0.5 absorbance units (A.U.) was considered the threshold (dotted horizontal line). (**b**) The immune profile of CAIP-specific T helper cells, isolated from the human atherosclerotic plaques, is shown. Carotid plaques were obtained by endoarterectomy from 8 CAIP-seropositive patients with atherosclerotic arteriopathy. CAIP-specific T cell clones derived from human atherosclerotic lesions were stimulated with or without CAIP, in presence of autologous antigen-presenting cells. IFN-γ, IL-4, IL-17 production was measured in culture supernatants. CD4^+^ clones able to produce IFN-γ, but not IL-4, were classified as Thl; CD4^+^ clones producing both IFN-γ and IL-17 were classified as Th1/Th17; CD4^+^ clones producing both IFN-γ and IL-4 were classified as Th0. In absence of CAIP stimulation the levels of IFN-γ, IL-4, IL-17 were consistently <7 pg/ml.

As the only evidence supporting CAIP production by Hc derives from a proteomic analysis performed on the bacterium *in vitro*^33^, we first verified whether Hc actually releases CAIP in infected hosts, considering the presence of protein-specific antibodies in the blood as a marker. Given that Hc can invade vascular tissues, we hypothesised that CAIP might play a role in atherosclerosis pathogenesis. Therefore, we developed an ELISA assay using a recombinant CAIP as antigen to investigate a cohort of patients with carotid atherosclerosis lesions, without verifying *a priori* their infectious state. Of the 71 patients included in our evaluation, 31 (43.6%) showed circulating antibodies specific for CAIP (Fig. 1a).

However, CAIP is very close to HP-NAP produced by *H. pylori,* as the two proteins have 79% of homology and 58% of identity (see Supplementary Fig. S1). Accordingly, a rabbit polyclonal antiserum raised against HP-NAP^31^ revealed CAIP in immunoblot (see Supplementary Fig. S1). Hence, considering the high incidence of *H. pylori* infection, the chance that the serum of atherosclerotic patients could recognize CAIP because of a cross-reactivity of their anti-HP-NAP antibodies was not negligible. Therefore, we screened all the patients for the presence of *H. pylori*-spcific IgG and found that 12 were *H. pylori*-infected among the 31 subjects whose serum gave an optical density (O.D.) higher than 0.5 (set as cut-off) when tested against CAIP. A cautious estimate of the percentage of atherosclerotic patients with a current or previous infection by Hc ranges between 26.7% (19 out of 71 subjects) and 43.6% (31 out of 71 subjects). In contrast, among the 38 healthy subjects that we studied as control, only 2, negative for *H. pylori* infection, showed an O.D. higher than 0.5 when tested against CAIP (5.2%). Hence, the presence of CAIP-specific antibodies in these subjects substantiates the view that the antigen is released by Hc in infected hosts.

### Atherosclerotic patients have CAIP-specific Th1 lymphocytes

The evidence that a sizable proportion of atherosclerotic patients have serum antibodies against CAIP, prompted us to search for T cells that were specific for the antigen in atherosclerotic lesions. *In vivo*-activated plaque-infiltrating T cells isolated from the carotid atherosclerotic lesions of 8 patients with anti-CAIP antibodies were expanded *in vitro* in IL-2–conditioned medium, subsequently cloned and studied for their phenotypic and functional profile^35^.

A total of 346 CD4^+^ and 83 CD8^+^ T-cell clones were obtained from the plaques. For each patient, CD4^+^ and CD8^+^ plaque-derived T-cell clones were assayed for proliferation in response to CAIP. None of the CD8^+^ T-cell clones showed proliferation to CAIP. In contrast, 34 (9.8%) of the 346 CD4^+^ T-cell clones generated from plaque-infiltrating T cells proliferated significantly in response to CAIP.

Upon antigen stimulation with CAIP, 26 (76.5%) of the 34 CAIP-specific CD4^+^ T-cell clones secreted IFN-γ, 5 (14.7%) clones produced both IL-17 and IFN-γ, and 3 clones (8.8%) produced IFN-γ and IL-4 (Fig. 1b). At variance, stimulation with tetanus toxoid did not induce any cytokine production in CAIP-specific clones (data not shown).

Taken together, these findings provide evidence that CAIP is an antigen produced by Hc in infected hosts and that CAIP-specific T cells, which mainly display a pro-inflammatory Th1 phenotype, infiltrate the atherosclerotic lesions of Hc-positive patients.

### CAIP has the dodecameric structure of the Dps-like family members

**Figure 2:**
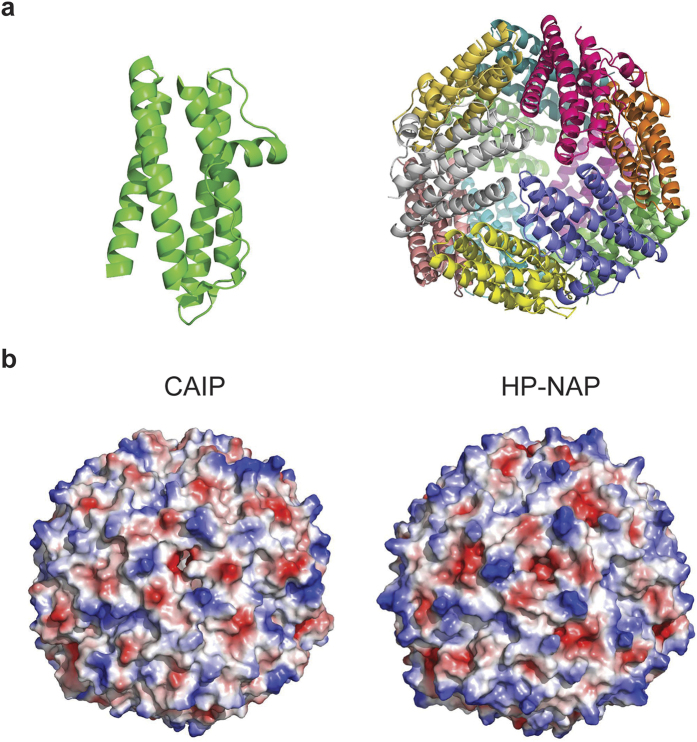
CAIP crystal structure. (**a**) Cartoon view of CAIP monomer (left) and dodecamer (right). Each subunit is shown in a different color. One of the three-fold molecular axes is in the center of the structure, perpendicular to the plane of the paper. (**b**) Qualitative surface electrostatic potential of CAIP (left) and HP-NAP (right). Positive charges are in blue, negative in red. A different charge distribution on the surface of the two proteins is evident.

We next crystalized CAIP and characterized its structure. As shown in Fig. 2a, CAIP presents the classical quaternary organization of all the other members of the Dps-like family^36^: a dodecameric shell of over 90 Å in diameter, formed by the twelve subunits arranged with a 32 symmetry. CAIP monomer displays the classical fold of Dps from *E. coli*, the structural prototype of the family^37^, namely a four-helix bundle, where the second α-helix is connected to the third one through a long stretch of 25 residues; in the middle of the latter a short α-helix is present. The superposition of the equivalent Cα‘s of CAIP to HP-NAP^38^ indicates that the two proteins are very similar. Inside the nearly spherical shell formed by the twelve subunits there is an internal cavity of over 40 Å in diameter, where in the other Dps-like proteins iron is stored. As in all the members of the Dps-like family, 12 iron binding sites are present in the putative ferroxidases centres, as detailed in Supplementary Information.

Significant differences in the charge distribution on the surface are evident between CAIP and HP-NAP, despite their structural similarity, as shown by the qualitative electrostatic charge distribution illustrated in Fig. 2b.

### Recombinant CAIP drives macrophage polarization towards the pro-inflammatory M1 profile

The most abundant cells that accumulate in atherosclerotic plaques are monocyte-derived macrophages^2^. The pro-inflammatory M1 macrophages may increase plaque vulnerability, whereas the pro-resolution M2 macrophages may increase plaque stability^39^.

Based on the evidence that CAIP drives a pro-inflammatory Th1 immune profile, we asked whether CAIP could lead to an unresolved inflammatory condition, also by interfering with the switch from pro-inflammatory to anti-inflammatory mediators that characterize the resolution phase.

To address this issue we investigated the impact of recombinant CAIP (produced in *Bacillus subtilis* and free of Gram-positive and Gram-negative contaminants, see Supplementary Figs S2 and S3) on human monocyte-derived macrophages differentiated towards the M1 or the M2 profile, given that both populations are observable in human atherosclerosis^40^.

**Figure 3:**
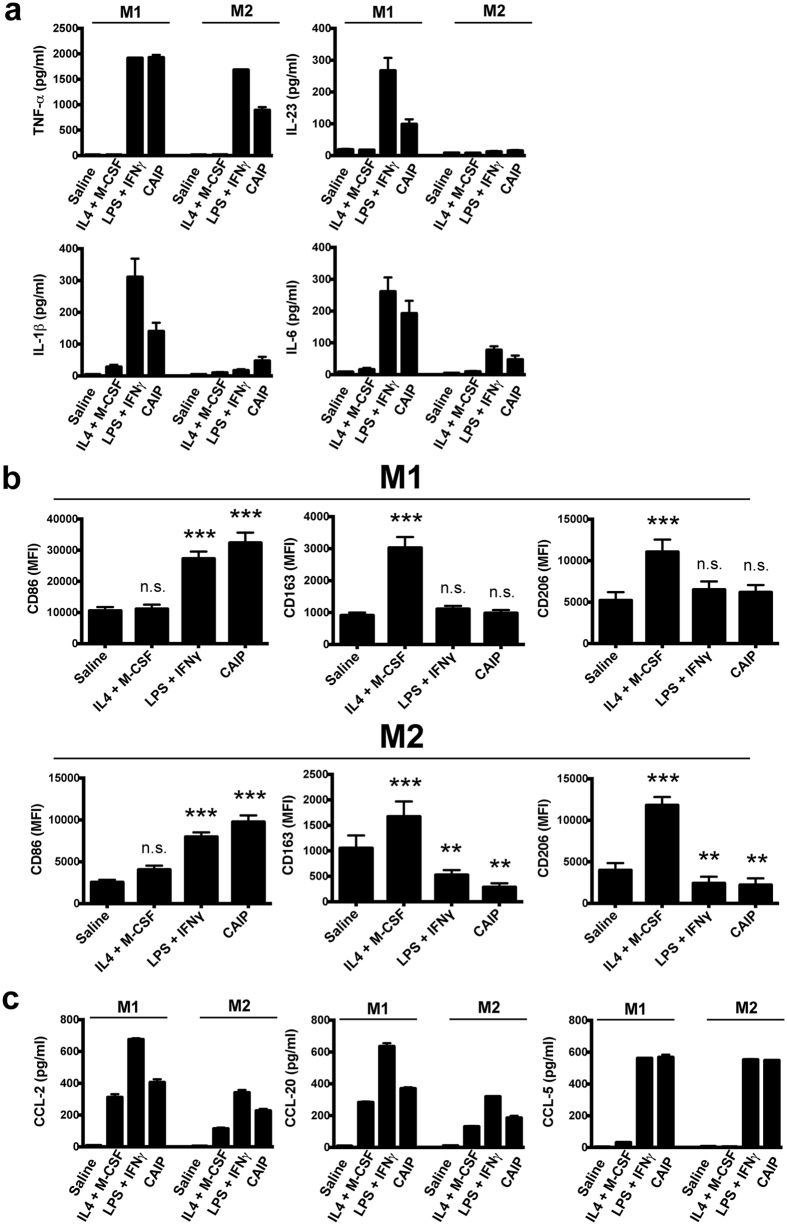
CAIP promotes the activation of macrophages and their polarization towards the M1 phenotype. Monocytes were differentiated for 6 d into M1 or into M2 macrophages and subsequently treated for 24 h with saline, IL-4 + M-CSF, LPS + IFN-γ or CAIP. (**a**) TNF-α, IL-23, IL-1β and IL-6 protein content in the culture supernatants. Data are expressed as mean value ± S.D. of 3 independent experiments performed with 3 different cell preparations. (**b**) Expression of CD86, CD163 and CD206 analysed by flow cytometry. Data are shown as Mean Fluorescence Intensity (MFI) ± S.D. of 3 independent experiments performed with 3 different cell preparations. Significance was determined by Student’s *t*-test versus saline-exposed cells. **p < 0.01; ***p < 0.001. (**c**) Levels of CCL-2, CCL-20 and CCL-5 in the culture supernatants quantified by specific ELISA assays. Data are expressed as mean value ± S.D. of 3 independent experiments performed with 3 different cell preparations.

Data showed that CAIP activates GM-CSF-differentiated M1 macrophages to release the pro-atherogenic cytokines TNF-α, IL-23, IL-1β and IL-6 (Fig. 3a). As expected, neither saline (vehicle of CAIP), nor the combination of the two M2-polarizing cytokines, IL-4 plus M-CSF, elicited any relevant effect. Noteworthy, CAIP induced the production of the pro-inflammatory mediators also in M-CSF-differentiated M2 macrophages, albeit to a lesser extent than in M1 cells (Fig. 3a), consistent with the plasticity of macrophages that can rapidly change phenotype and function in response to micro-environmental signals^41^. The evidence that, following the exposure to CAIP, both M1 and M2 macrophages increased the expression of CD86, alongside the down-modulated expression of the two classical M2 surface markers CD163 and CD206 (Fig. 3b), provided convincing support to the conclusion that CAIP promotes M1 macrophage polarization. Interestingly, even though HP-NAP of *H. pylori* is a pro-inflammatory antigen^31^, its impact on the profile of M1 and M2 macrophages was less pronounced than that of CAIP (see Supplementary Fig. S4).

The pro-inflammatory activity of CAIP was confirmed also by the finding that it stimulates M1 and M2 macrophages to release CCL-2, CCL-20 and CCL-5 (Fig. 3c). These chemokines are important in atherogenesis, because they recruit mononuclear cells from the circulation, thereby sustaining the inflammatory status^42^. Moreover, CCL-2 and CCL-20 promote the migration of Th17 lymphocytes that, together with Th1 cells, are crucial for the development of the atherosclerotic plaques^9^.

### CAIP promotes the accumulation of LDL in macrophages

**Figure 4:**
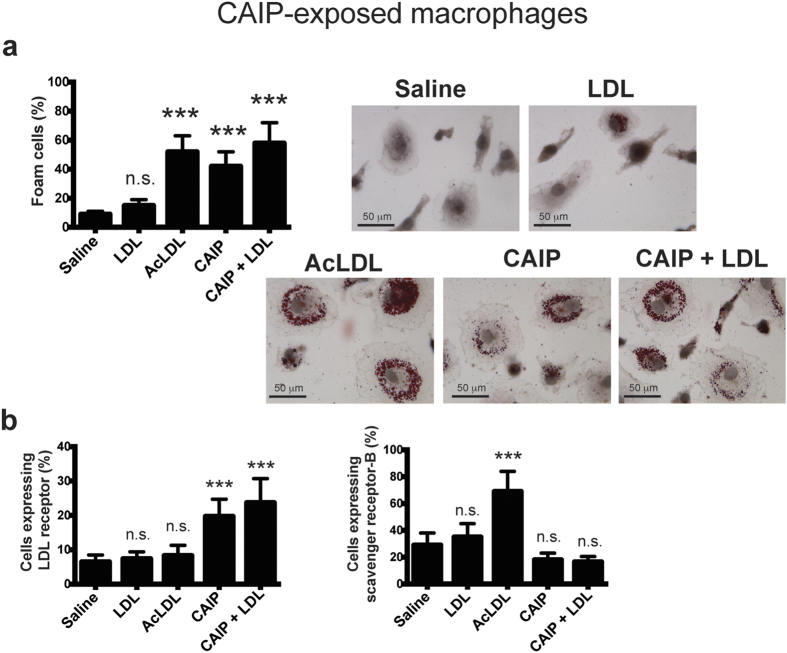
CAIP induces foam cell formation. (**a**) Macrophages treated 24 h with saline, LDL, AcLDL, CAIP or CAIP + LDL were stained with Oil Red O. Images are representative of one of 3 independent experiments performed with 3 different cell preparations. The number of foam cells was determined and expressed as percentage of total cells counted in 10 random fields. Data are expressed as mean value ± S.D. of 3 independent experiments performed with 3 different cell preparations. Significance was determined by Student’s *t*-test versus saline-exposed cells. ***p < 0.001. (**b**) Cells were analysed for the surface expression of LDL receptor and scavenger receptor-B by flow cytometry, after a 24-h treatment. Data are shown as mean percentage of positive cells ± S.D. of 3 independent experiments performed with 3 different cell preparations. Significance was determined by Student’s *t*-test versus saline-exposed cells. ***p < 0.001.

Lipoprotein uptake by macrophages leading to formation of foam cells is thought to be one of the earliest pathogenic events in the nascent plaque^3^. Since Hc induces macrophage foam cell formation^26^, we examined the possibility that CAIP might promote lipid accumulation in macrophages. As shown in Fig. 4a, CAIP stimulated the formation of lipid droplets in macrophages exposed to unmodified human low-density lipoproteins (LDL). Notably, the exposure of macrophages to CAIP alone also induced the storage of lipids in the cytoplasm, although to a lesser extent than in the presence of LDL. This probably reflects the uptake of the LDL cholesterol available from the 10% serum used to complete the cell culture medium^26^. The efficient uptake of LDL paralleled an increase in LDL receptor (LDLr) expression (Fig. 4b), consistent with previous studies showing that LDLr mediates the accumulation of unmodified LDL in macrophages^43^,^44^. The expression of the scavenger receptor class B (SR-B), involved in the uptake of modified LDL, was up-regulated in cells exposed to acetylated-LDL (AcLDL)^45^, but was not affected by the treatment with CAIP (Fig. 4b).

Collectively, these data suggest that CAIP could contribute to foam cell accumulation in the atherosclerotic plaques because it promotes LDL uptake by macrophages. Notably, this activity is not a feature that characterizes the proteins encoded by napA (IN ITALIC FONT) genes of *Helicobacter spp*.; indeed, HP-NAP of *H. pylori* did not induce the formation of foam cells from macrophages (see Supplementary Fig. S4).

### CAIP activates endothelial cells

At atherosclerotic lesion-prone sites the up-regulation of cell adhesion molecules and chemokines mediate the recruitment of circulating monocytes that, in turn, lead to chronic inflammation and progression of atherosclerosis^46^. Accordingly, the adhesion molecules E-selectin and VCAM-1 have been demonstrated to be key elements in the development of atherosclerotic lesions^2^.

**Figure 5:**
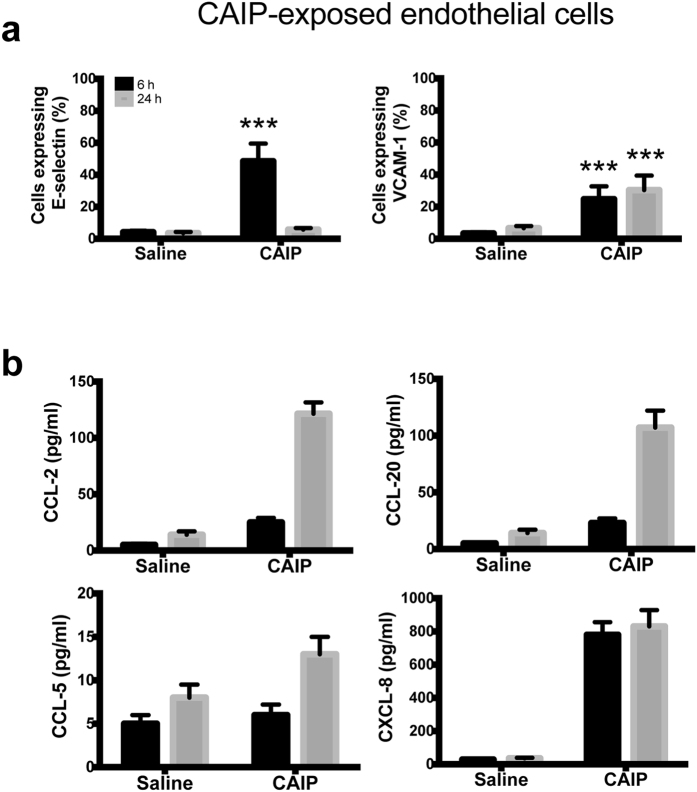
CAIP induces expression of adhesion molecules and chemokines in endothelial cells. Endothelial cells exposed to saline or CAIP for 6 and 24 h. (**a**) Surface expression of E-selectin and VCAM-1, as determined by flow cytometry. Data are expressed as mean percentage of positive cells ± S.D. of 3 independent experiments performed with 3 different cell preparations. Significance was determined by Student’s *t*-test versus saline-exposed cells at each time point. ***p < 0.001. (**b**) Levels of CCL-2, CCL-20, CCL-5 and CXCL-8 accumulated in the culture supernatants quantified by specific ELISA assays. Data are expressed as mean ± S.D. of 3 independent experiments performed with 3 different cell preparations.

To investigate whether CAIP could alter the production of cell adhesion molecules and chemokines in endothelial cells, we exposed Human Umbilical Vein Endothelial Cells (HUVECs) to the recombinant bacterial protein. We found that CAIP up-regulated E-selectin and VCAM-1 expression (Fig. 5a). Both adhesion molecules showed a peak of expression after a 6 h stimulation. E-selectin expression dropped down at 24 h, while VCAM-1 expression remained high longer.

Next, by testing the release of chemokines by endothelial cells exposed to CAIP we found a time-dependent accumulation of CCL-2 and CCL-20 in the culture supernatant. We could not find any production of CCL-5 by HUVECs probably because they are not the best endothelial cell model for evaluating CCL-5 production since they have been already reported not to respond to other pro-inflammatory stimuli^47^. Noteworthy, CAIP stimulated the release of CXCL-8 (Fig. 5b), a chemokine mainly known as neutrophil chemoattractant but shown to play an important role in monocyte migration into the sub-endothelial space in the early phase of atherosclerosis^48^.

Taken together, our data indicate that CAIP up-regulates the expression of both adhesion molecules and chemokines, thus recruiting mononuclear cells and contributing to the chronic inflammatory process that characterizes the atherosclerotic plaques.

### CAIP drives the differentiation of monocytes towards macrophages

The continuous recruitment of circulating monocytes to the inflamed tissue of the plaques, and their subsequent differentiation into macrophages, is a hallmark of the atherosclerotic process^49^.

**Figure 6:**
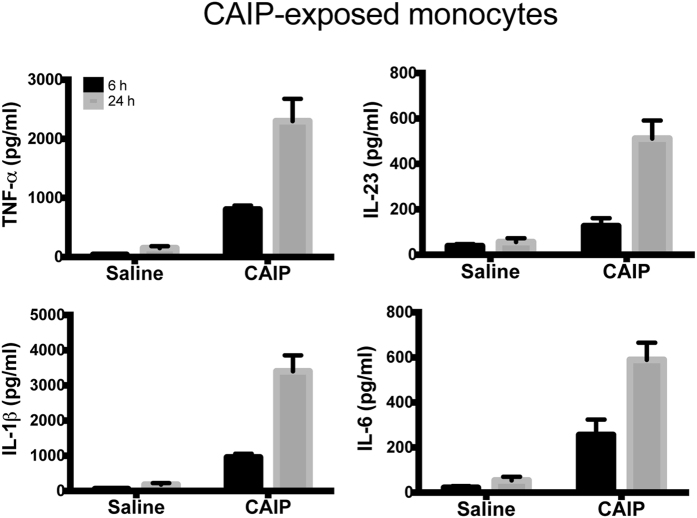
CAIP induces TNF-α, IL-23, IL-1β and IL-6 production in monocytes. Cells were exposed to saline or CAIP for 6 and 24 h. Culture supernatants were collected and the cytokine content was determined by ELISA. Data are expressed as mean value ± S.D. of 3 independent experiments performed with 3 different cell preparations.

**Figure 7:**
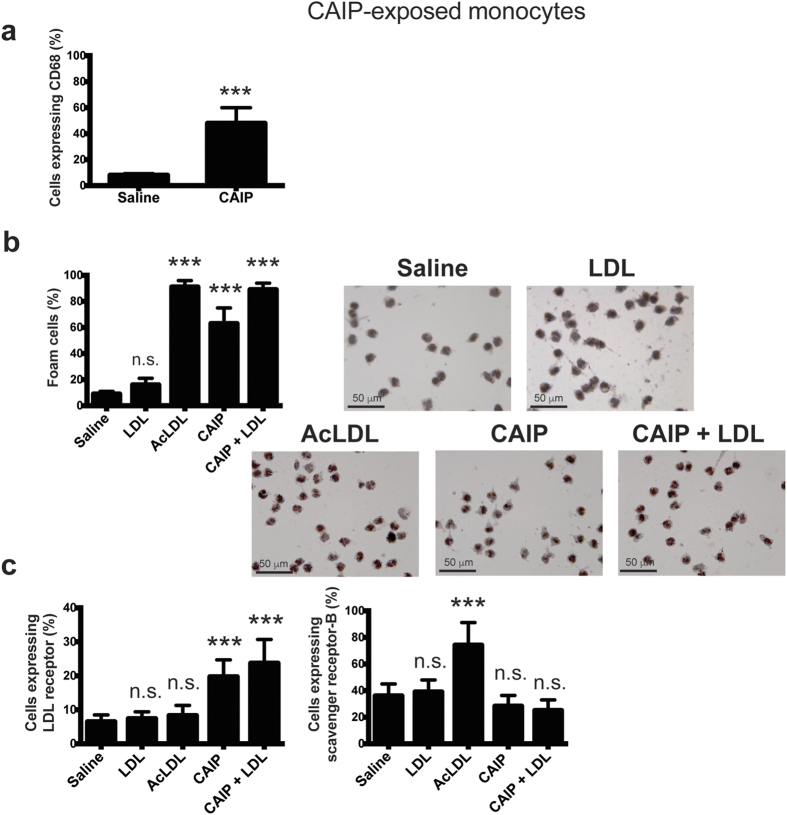
CAIP promotes the differentiation of monocytes into macrophage-derived foam cells. (**a**) Expression of CD68 on monocytes exposed to saline or CAIP for 24 h. Data are shown as mean percentage of positive cells ± S.D. of three independent experiments from three different donors. Significance was determined by Student’s *t*-test versus saline-exposed cells. ***p < 0.001. (**b**) Monocytes treated 24 h with saline, LDL, AcLDL, CAIP or CAIP + LDL were stained with Oil Red O. Images are representative of one of 3 independent experiments performed with 3 different cell preparations. The number of foam cells was determined and expressed as percentage of total cells counted in 10 random fields. Data are expressed as mean value ± S.D. of 3 independent experiments performed with 3 different cell preparations. Significance was determined by Student’s *t*-test versus saline-exposed cells. ***p < 0.001. (**c**) Cells were analysed for the surface expression of LDL receptor and scavenger receptor-B by flow cytometry, after a 24-h treatment. Data are shown as mean percentage of positive cells ± S.D. of 3 independent experiments performed with 3 different cell preparations. Significance was determined by Student’s *t*-test versus saline-exposed cells. ***p < 0.001.

Given the documented pro-inflammatory activity of CAIP, we moved to evaluate whether it might activate also the newly recruited monocytes. Human monocytes exposed to CAIP secreted TNF-α, IL-23, IL-1β and IL-6 (Fig. 6). We also noticed that, after a 24 h-stimulation with CAIP, monocytes harboured morphological change, thus resembling macrophages (data not shown). Hence, monocytes treated with CAIP were evaluated for the expression of the macrophage marker CD68. The results showed a strong increase in CD68 expression after a 24 h-exposure to the antigen (Fig. 7a). Moreover, CAIP-treated monocytes engulfed unmodified LDL (Fig. 7b); this activity paralleled the up-regulation of LDLr, whereas the scavenger receptor SR-B remained unaffected (Fig. 7c). As in the case of macrophages, the exposure of monocytes to HP-NAP in presence of unmodified LDL did not result in the accumulation of lipid droplets in the cytoplasm (see Supplementary Fig. S4). This is in agreement with the evidence that HP-NAP of *H. pylori* stimulates monocytes to differentiate into dendritic cells rather than into macrophages^31^.

Collectively, our data further support the notion that CAIP acts as a strong pro-inflammatory stimulus. As such, the antigen is expected to be one of the major determinants produced by Hc responsible for the state of chronicity of the inflammation at the atherosclerotic lesions.

### Macrophage activation by CAIP results from the engagement of a G_i_ protein-coupled receptor and involves the p38/ERK signal transduction pathway

Seeking for a potential cell receptor for CAIP, considering the similarity between CAIP and HP-NAP, we hypothesized that the former might engage a G-protein coupled receptor as reported for HP-NAP^50^. To address this possibility, we took advantage of pertussis toxin (PTX) which, by ADP-ribosylating the Gα subunits of heterotrimeric G proteins of the Gi and Go subtypes, blocks the signalling pathway elicited by seven-spanning transmembrane receptors to which they are coupled^51^.

**Figure 8:**
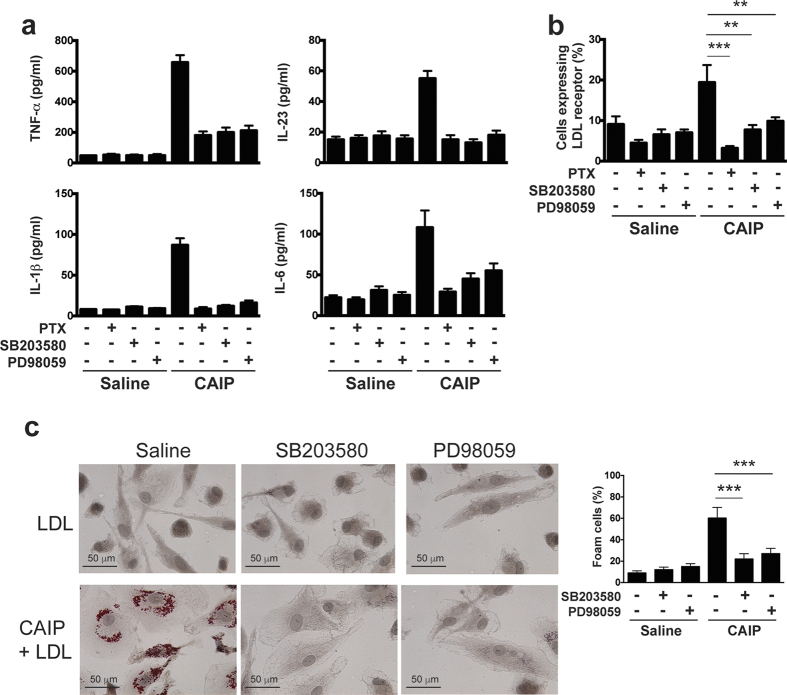
CAIP activates a Gi protein-coupled receptor in macrophages. Macrophages were either untreated or pre-incubated 16 h with PTX or 30 min with SB203580 or PD98059 and then treated with saline or CAIP for 24 h. (**a**) TNF-α, IL-23, IL-1β and IL-6 protein content in the culture supernatants. Data are expressed as mean value ± S.D. of 2 independent experiments performed with 2 different cell preparations. (**b**) Cells were analysed for the surface expression of LDL receptor by flow cytometry. Data are shown as mean percentage of positive cells ± S.D. of 2 independent experiments performed with 2 different cell preparations. Significance was determined by Student’s *t*-test. **p < 0.01 and ***p < 0.001. (**c**) Macrophages were stained with Oil Red O. Images are representative of one of 2 independent experiments performed with 2 different cell preparations. The number of foam cells was determined and expressed as percentage of total cells counted in 10 random fields. Data are expressed as mean value ± S.D. of 2 independent experiments performed with 2 different cell preparations. Significance was determined by Student’s *t*-test. ***p < 0.001.

Figure 8 shows that PTX is a powerful inhibitor of the effects of CAIP on macrophages: it interferes with CAIP-induced cytokines release and expression of LDLr on cell surface (Fig. 8a,b), indicating that the effects of CAIP on macrophages are mediated by a Gi-coupled receptor.

The activation of Gi decreases the adenylate cyclase activity, but in parallel activates a signalling pathway that involves the MAPKs p38 and ERK^52^,^53^. Consistent with the effects observed with PTX addition, we found that the p38 inhibitor SB203580 prevents the effects of CAIP on macrophages. Similar results were obtained upon the inhibition of the kinase ERK by PD98059 (Fig. 8a–c). As expected, the inhibition of the Gi-coupled signalling pathway completely abrogated the effects of CAIP also on monocytes (data not shown).

Interestingly, by assessing the effects of CAIP on endothelial cells, we found that PTX increased, rather than reduced the expression of VCAM-1 induced by CAIP (Fig. S5). This result suggests that in endothelial cells CAIP activates two independent pathways leading to opposite effects: a stimulatory pathway that is responsible for the expression of VCAM-1 and a Gi-dependent pathway that negatively regulates the former. Once the inhibitory pathway is blocked, the stimulatory one takes over, resulting in a greater expression of the adhesion molecule.

Finally, the observation that, unlike VCAM-1, the expression of E-selectin induced by CAIP was not affected by PTX, suggests that this adhesion molecule is regulated by CAIP through a different pathway that does not involve Gi proteins (Fig. S5).

## Discussion

The enterohepatic bacterium *Helicobacter cinaedi*^13^,^14^ isolated from immunocompromised patients with bacteremia, cellulitis, and septic arthritis^15^,^54^, recently gained a spotlight on the stage of atherogenesis following its isolation from an immunocompetent patient with myopericarditis^22^. Subsequently, Hc was cultured from surgically removed aneurysms characterized by severe atherosclerosis and inflammation^23^ and bacterial antigens were found in macrophages of atherosclerotic aortic tissues^25^. The bacterium was shown to trigger the differentiation of monocytes into macrophages releasing pro-inflammatory cytokines and to cause the formation of foam cells^26^. This evidence highlighted that the pro-atherogenic potential of Hc relies on its ability to promote and maintain an inflammatory status. Most importantly, the oral administration of Hc to hyperlipidemic mice resulted in the promotion of atherosclerosis^26^. Our finding that at least 27% of atherosclerotic patients have antibodies specific for CAIP further supports the notion of an involvement of the bacterium in atheroma formation.

Based on the complete genome sequence of Hc, the bacterium is predicted to produce several virulence factors, such as a cytolethal distending toxin (CDT), an alkyl hydroperoxide reductase, and a neutrophil activation protein. The former is the only virulence factor studied so far^28^ and because of the homology to CDT from other bacteria such as *Campylobacter jejuni* and *Helicobacter hepaticus*, Hc CDT was suggested to operate in immune modulation and persistent bacterial colonization^26^,^55^,^56^. However, the evidence that a *cdt* mutant of Hc increases as the wild-type strain the expression of TNF-α, IFN-γ and iNOS in the coecum of infected mice^57^, suggests that other bacterial factors than CDT could tune the function of immune cells in infected hosts.

With this study we have identified the protein encoded by the napA (IN ITALIC FONT) gene as a novel Hc antigen, which we named Cinaedi Atherosclerosis Inflammatory Protein (CAIP), that possesses a strong immuno-modulatory activity. CAIP activates M1 macrophages to release inflammatory cytokines and redirects the pro-resolution M2 macrophages towards the pro-inflammatory profile, thus interfering with the resolution of inflammation. The production of cell adhesion molecules and chemokines in endothelial cells exposed to CAIP is expected to exacerbate the process, because of the continuous recruitment of leukocytes.

CAIP possesses a dodecameric structure similar to that of the other members of the Dps-like family, key factors involved in the protection of prokaryotic cells from oxidative damage^58^, and in particular to that of HP-NAP, a major antigen produced by *H. pylori*, whose impact on the function of immune cells is well documented^31^,^32^. However, the two proteins have an overall identity of 58% and possess a different charge distribution on the surface, which may account for their different properties. In fact, while the exposure of human monocytes to HP-NAP leads to their maturation towards dendritic cells^31^, CAIP-treated monocytes become macrophages and internalize LDL, thereby transforming into foam cells. Neither monocytes nor macrophages accumulate lipids following the exposure to HP-NAP.

CAIP activates macrophages upon engagement of a Gi protein-coupled receptor; consistent with the notion that ERK and p38 are activated by a Gi protein-elicited pathway^52^, its effects are prevented when either of these MAPKs is inhibited. Interestingly, when we assessed whether CAIP affects endothelial cells through this pathway, we observed that VCAM-1 expression increased in cells that were incubated with PTX, prior the stimulation with CAIP compared to cells exposed to CAIP alone. This result, which accords well with the data of Sadeghi and colleagues^59^ showing that IL-1-induced ICAM expression on HUVECs was potentiated by PTX treatment, suggests an inhibitory role of a Gi protein in the process. Therefore, as already suggested for IL-1, CAIP might trigger a dual response in endothelial cells: one stimulatory and a G-protein coupled inhibitory one. Blockage of the latter by PTX would leave the stimulatory pathway to be unopposed, leading to enhanced expression of the adhesion molecule. Unlike VCAM-1, E-selectin induced by CAIP was not affected by PTX, suggesting a different mechanism of regulation for this adhesion molecule that does not involve Gi proteins. Therefore, the signalling pathway triggered by CAIP in endothelial cells appears to be far more complex than in macrophages and deserving further investigation.

Our results indicating that CAIP-specific T cells accumulate in the atherosclerotic plaques of Hc-infected patients support the notion that CAIP is able both to modulate the adaptive immune response and to contribute to establishing the typical Th1/Th17 inflammatory atherosclerosis signature.

Collectively, our data suggest that CAIP might be one of the bacterial factors that contribute to the pathogenesis of atherosclerosis associated to Hc infection and that preventing and eradicating the bacterial infection could reduce the incidence of atherosclerosis.

## Methods

### Ethics Statement

All the experimental protocols were approved by the ethical committee of the Department of Experimental and Clinical Medicine, University of Florence, Italy. All the investigations were carried out in accordance with relevant guidelines and regulations. Written informed consent was obtained from all the subjects. Peripheral blood mononuclear cells utilized in this study derived from buffy coats obtained from healthy blood donors. Umbilical cords were obtained from full-term healthy pregnant women.

### Expression and purification of recombinant CAIP

The *napA* gene was synthetized by GeneArt (Invitrogen), based on the sequence HCN_0606 (URL: http://www.ncbi.nlm.nih.gov/nuccore/AP012344). The recognition sites for the restriction enzymes SacI and HindIII were added at the 5′ end and 3′-end of the sequence, respectively. The gene was digested, inserted into the expression vector pSM214G and cloned and expressed in the *B. subtilis* strain SMS 118 as reported elsewhere^60^. *B. subtilis* strain SMS 118 containing the plasmid pSM214G-*napA* was grown for 15 h in YT medium plus 15 μg/ml of chloramphenicol. After centrifugation, cells were resuspended in 30 mM Tris HCl, pH 7.8, and lysed through 3 French press passages. Debris were removed by centrifugation, and 70% ammonium sulfate was added to the supernatant.

After centrifugation at 32,000 g, the supernatant was dialyzed and loaded onto a pre-packed anion-exchange column (Mono Q fast-protein liquid chromatography column; GE Healthcare). CAIP was eluted in a range of 0.24–0.37 M NaCl. The fractions containing the protein were pooled, and CAIP was further purified by gel-filtration chromatography (Superdex 200 HR 10/30; GE Healthcare) with phosphate buffer (PBS), pH 7.8. Protein was concentrated using a Centricon ultrafiltration system (Millipore, Bedford, MA). The final product was checked for purity (above 98%) in a Coomassie brilliant blue–stained gel.

### CAIP-specific antibody assay in atherosclerotic patients and healthy subjects and evaluation of the *H. pylori* seropositivity of the same individuals

Serum samples were obtained from 71 patients (36 males and 35 females; age range 62–74) with carotid atherosclerosis and from 38 sex- and age-matched healthy control subjects.

Serum samples were diluted 1:1000 before adding to the wells in a 96-well plate previously coated with purified recombinant CAIP (1 μg/well). Horseradish peroxidase–conjugated anti-human IgG subclass antibody was added to each well, and colour was developed with TMB. Absorbance was read at 450 nm. According to Classen and colleagues^61^, for determining the ELISA’s cut-off we adopted the general formula: cut-off = *a* · X + *f* · S.D., where X is the mean and S.D. the standard deviation of independent negative control readings, and *a* and *f* two multipliers. Although *a* is usually fixed at 1 and *f* at 3, in our analysis, we prudentially considered 5 as value for *f*, thus our cut-off (=mean + 5 times the standard deviation) was set at 0.5 O.D.

The same subjects were tested for seropositivity of antibodies against *H. pylori* by a specific ELISA kit (Biohit HealthCare), according to the manufacturer’s instruction.

### Purification of the cells and treatments

Monocytes from healthy donors were prepared as described previously^31^. Briefly, PBMC from healthy donors were isolated by centrifugation on Ficoll-Paque solution. Cells were laid on a cushion of Percoll 46% v/v solution in RPMI 1640 supplemented with 10% FCS, 50 μg/ml gentamicin, and 4 mM HEPES. Monocytes were harvested, resuspended in medium containing 2% FCS, and further separated from contaminating lymphocytes by adherence (1 h at 37 °C) to plastic wells. Adherent monocytes were extensively washed with medium to remove residual non-adherent cells. Monocytes were then cultured in RPMI 1640 10% FCS, 50 μg/ml gentamicin and 4 mM HEPES. 2 × 10^6^ monocytes, seeded in 24-well plates, were exposed to 20 μg/ml CAIP or saline (PBS) in RPMI 1640 containing 10% FCS.

For macrophage differentiation, 5 × 10^5^ monocytes, seeded in 24-well plates, were cultured in RPMI 20% FBS in the presence of 100 ng/ml GM-CSF (M1) or 100 ng/ml M-CSF (M2) for 6 d. Medium was partially replaced after 3 d. Differentiated macrophages were treated with 20 μg/ml CAIP, 20 μg/ml HP- NAP, 100 ng/ml LPS + 20 ng/ml IFN-γ (M1-polarizing cocktail), 10 ng/ml M-CSF + 20 ng/ml IL-4 (M2-polarizing cocktail) or saline, in RPMI 1640 containing 10% FCS.

Human umbilical vein endothelial cells (HUVECs) were isolated after collagenase treatment of cords and cultured as described elsewhere^62^. Briefly, the umbilical vein was cannulated and perfused with sterile PBS to wash out the blood and allowed to drain. Twenty ml of collagenase 5 U/ml were infused into the vein and the cords were incubated 15 min at 37 °C. A perfusion with 30 ml of wash buffer (RPMI1640, 20% FBS, L-glutamine 2 mM, penicillin 100 U/ml and streptomycin 100 μg/ml) permitted to harvest the cells that, after centrifugation, were resuspended in complete medium (M199, 20% FBS, heparin 50 μg/ml, EGF 20 ng/ml, L-glutamine 2 mM, penicillin 100 U/ml and streptomycin 100 μg/ml) and grown in tissue culture plates (Costar) coated with 2% endotoxin-free gelatin. Cells (used at passage 2–5) were exposed to 20 μg/ml CAIP or saline in M199 complete medium.

When indicated cells were pre-incubated with 100 ng/ml PTX (Tocris Bioscience) for 16 h, with 10 μM SB203580 (Sigma) or with 50 μM PD98059 (Calbiochem) for 30 min, before CAIP stimulation.

### Detection of TNF-α, IL-23, IL-1β, IL-6, CCL-2, CCL-20, CCL-5 and CXCL-8 in culture supernatants

Culture supernatants from monocytes, macrophages, and HUVECs were collected for quantification of cytokine protein levels in the supernatants, which was done by ELISA, using specific kits (eBiosciences), according to manufacturer’s instructions.

### Flow cytometry

Phenotypic analysis of macrophages was performed by flow cytometry. Cells were harvested from culture plates using 5 mM Na-EDTA in PBS pH 7.5 and incubated for 15 min at RT with 5% human serum to saturate Fc receptors.

5 × 10^5^ cells were stained with a monoclonal antibody anti-human CD86-PE (eBiosciences), anti-human CD163-PerCP-Cy5.5 and anti-human CD206-APC (BD Biosciences). Cells were washed, fixed and permeabilized for 20 min at 4 °C (Cytofix/Cytoperm Kit; BD Biosciences) and further stained with a monoclonal antibody anti-CD68-FITC. Cells were washed, resuspended in FACS buffer (PBS, 1% BSA) and analysed by a six-colour FACSCanto II (Becton Dickinson). Forward and side scatter light were used to identify cell populations. Values were expressed as the ratio of the mean fluorescence intensity (MFI) of the marker of interest over the MFI of the isotype control.

For evaluating the expression of LDL receptors, a rabbit polyclonal antibody anti-human LDL receptor (LDLr) (Abcam) and a monoclonal antibody anti-human scavenger receptor (SR)-B-PE-Cy7 (eBiosciences) were applied. LDLr was revealed by a secondary goat anti-rabbit IgG-FITC. Monocytes were labelled for CD68, LDLr and SR-B as macrophages.

For evaluating the expression of adhesion molecules on HUVECs, cells were harvested and saturated as above. E-selectin (CD62E) and VCAM-1 (CD106) were revealed by mouse anti-CD62E-FITC and CD106-PE (eBiosciences), respectively. All data were analysed using FlowJo software, version 10.3 (Tree Star Inc.). Values were expressed as mean percentage of positive cells.

When indicated cells were pre-incubated with 100 ng/ml PTX for 16 h, with 10 μM SB203580 or with 50 μM PD98059 for 30 min, before CAIP stimulation.

### Foam cell formation

5 × 10^5^ monocytes, differentiated into M2, or 2 × 10^6^ monocytes, were plated on glass cover slips in a 24-well plate. Cells were exposed for 24 h to 20 μg/ml CAIP, 20 μg/ml CAIP + 30 μg/ml LDL, 20 μg/ml HP-NAP, 20 μg/ml HP- NAP + 30 μg/ml LDL, 30 μg/ml AcLDL or saline. Cells were fixed in 4% buffered formalin for 10 min, washed in distilled water, rinsed in 60% isopropanol and stained with 0.3% Oil Red O for 15 min. Stained cells were then cleaned in 60% isopropanol and counterstained with haematoxylin. Images were obtained using a Leica DMR microscope with 63x magnification. Quantification of the lipid accumulation was performed with Fiji software, as reported elsewhere^63^: cells with more than 10 lipid droplets were defined as foam cells and the percentage of foam cells formed in each condition was determined according to a previously described method^63^.

When indicated cells were pre-incubated with 100 ng/ml PTX for 16 h, with 10 μM SB203580 or with 50 μM PD98059 for 30 min, before CAIP stimulation.

### Generation of T cell clones from atherosclerotic plaques and analysis of their profile

Fragments of carotid atherosclerotic plaques were obtained by endoarterectomy from eight CAIP-seropositive patients (4 males, 4 females, mean age 64; range 57–69 y) with atherosclerotic arteriopathy, and were cultured for 7 d in RPMI 1640 supplemented with IL-2 (50 U/ml) to expand *in vivo*-activated T cells. Specimens were then disrupted, and single T cells were cloned under limiting dilution, as described^9^. Anti-human CD3-PERCP-Cy5.5, anti-human CD4-FITC and anti-human CD8-PE (BD Biosciences) were used for cell surface marker analysis of T-cell clones. A Becton Dickinson LSR-BDII cytofluorimeter was used to perform the analysis. Clones were screened for responsiveness to CAIP and tetanus toxoid antigens by measuring [3H]thymidine uptake after 60 h of co-culture with irradiated autologous mononuclear cells in the presence of medium, CAIP (10 μg/ml), and tetanus toxoid (0.5 μg/ml), as reported elsewhere (Benagiano et al., 2012). At 16 h before harvesting, 0.5 μCi of [3 H]thymidine were added, and radionuclide uptake was measured in a β-counter. The mitogenic index (MI) was calculated as the ratio between mean values of cpm obtained in stimulated cultures and those obtained in the presence of medium alone. MI > 5 was considered as positive. To assess the cytokine production of CAIP-specific clones on antigen stimulation, 5 × 10^5^ T cell blasts of each clone were co-cultured for 48 h in 0.5 ml of medium with 5 × 10^5^ irradiated autologous peripheral blood mononuclear cells in the absence or presence of CAIP (10 μg/ml) or tetanus toxoid (0.5 μg/ml). At the end of culture period, duplicate samples of each supernatant were assayed for IFN-γ, IL-4, and IL-17 (R&D Systems, Abingdon, UK). CD4^+^ clones secreting IFN-γ were coded as Th1, clones producing both IL-17 and IFN-γ were categorized as Th1/Th17, whereas those secreting IFN-γ and IL-4 were coded as Th0.

### Statistical analysis

Data are reported as the mean ± SD. Student’s *t*-test was used for statistical analysis of the differences between experimental groups. P values less than 0.05 were considered significant.

## Additional Information

**How to cite this article**: D’Elios, M. M. *et al*. The *Helicobacter cinaedi* antigen CAIP participates in atherosclerotic inflammation by promoting the differentiation of macrophages in foam cells. *Sci. Rep.* **7**, 40515; doi: 10.1038/srep40515 (2017).

**Publisher's note:** Springer Nature remains neutral with regard to jurisdictional claims in published maps and institutional affiliations.

## Supplementary Material

Supplementary Information
