# Supplementary material for: The Helicobacter cinaedi antigen CAIP participates in atherosclerotic inflammation by promoting the differentiation of macrophages in foam cells
Source: Sci Rep. 2017 Jan 11;7:40515. doi: 10.1038/srep40515 (PMC5225449; doi:10.1038/srep40515)
Supplement: Supplementary Information [file srep40515-s1.pdf]

Supplementary information

The Helicobacter cinaedi antigen CAIP participates in atherosclerotic inflammation by promoting the differentiation of macrophages in foam cells

Mario Milco D'Elios, Francesca Vallese, Nagaja Capitani, Marisa Benagiano, Maria Lina Bernardini, Mirko Rossi, Gian Paolo Rossi, Mauro Ferrari, Cosima Tatiana Baldari, Giuseppe Zanotti, Marina de Bernard*, Gaia Codolo*

*Co-corresponding authors

Supplementary Figure Legends

Supplementary Figure S1. Homology and molecular mimicry between *Helicobacter cinaedi* CAIP and *Helicobacter pylor*i HP-NAP. (A) Multiple alignment performed with ClustalW software between CAIP and HP-NAP. * = identical amino acids; : = highly homologous amino acids; . = homologous amino acids. (B) 500 ng of CAIP and of HP-NAP were loaded in 4-12% SDS-PAGE and transferred to nitrocellulose. Proteins were revealed by a polyclonal antibody specific for HP-NAP[1]. Molecular weights (MW, expressed in kDa) are reported.

Supplementary Figure S2. CAIP preparation is free from Gram-positive contaminants. HEK-293 cells expressing human Toll-Like-Receptor 2 (hTLR2) were challenged with 20 μg/ml CAIP or with 1 μg/ml Pam3CSK4, as positive control. After 6 h, NF-κB activation (left panel) was measured using the luciferase reporter assay system, and normalized to that of saline-exposed cells, whose values were set at 1 arbitrary unit (A.U.). IL-8 production (right panel) was quantified after 18 h stimulation with the specified agonists. Data are the mean values ± S.D. obtained from three

independent experiments repeated twice. Significance was determined by Student's *t*-test versus saline-exposed cells. ***p < 0.001.

Supplementary Figure S3. The small LPS contamination does not affect the immune modulant activity of CAIP. (A) HEK-293 cells expressing human Toll-Like-Receptor 4 (hTLR4)/MD2/CD14 were challenged with 20 μg/ml CAIP or with 100 ng/ml *E. coli* LPS, as positive control. After 4 h, NF-κB activation (left panel) was measured using the luciferase reporter assay system, and normalized to that of saline-exposed cells, whose values were set at 1 A.U. IL-8 production (right panel) was quantified after 18 h stimulation with the specified agonists. Data are the mean values ± S.D. obtained from three independent experiments repeated twice. Significance was determined by Student's *t*-test versus saline-exposed cells. **p < 0.01; ***p < 0.001. (B) Monocytes were exposed to 20 μg/ml CAIP (boiled or not), to 100 ng/ml LPS (boiled or not) or saline (negative control) for 2 h and the expression of IL-12p40, IL-23p19, IL-1β, IL-6 and TNF-α was evaluated by RT-PCR. Data were normalized to an endogenous reference gene (GAPDH). Saline-treated cells were taken as reference and set as 1 A.U. (indicated by the dotted line) and the expression levels for treated cells were relative to the expression of negative control cells.

Supplementary Figure S4. CAIP and HP-NAP have a different impact on macrophages. (A) Expression of CD86, CD163 and CD206 on macrophages exposed to CAIP or HP-NAP for 24 h. Data are shown as Mean Fluorescence Intensity (MFI) ± S.D. of 3 independent experiments performed with 3 different cell preparations. (B) LDL uptake by macrophages and monocytes exposed for 24 h to CAIP or to HP-NAP. Foam cells were identified by Oil Red O staining. Images are representative of one of 3 independent experiments performed with 3 different cell preparations. Scale bar, 50 μm.

Supplementary Figure S5. CAIP activates a double pathway in endothelial cells. HUVECs were pre-incubated 16 h with 100 ng/ml PTX before the exposure to saline or CAIP for 6 and 24 h. Surface expression of E-selectin and VCAM-1 were determined by flow cytometry. Data are expressed as mean percentage of positive cells ± S.D. of 2 independent experiments performed with 2 different cell preparations. Significance was determined by Student's *t*-test. **$p < 0.01$.

Supplementary Table 1. Statistics on data collection and refinement

**Data set**	**Orthorhombic**
Wavelength	0.872899 Å
Space group	$P2_1$
Cell parameters (Å)	**a**=89.746, **b**=113.918, **c**=93.445, β=114.50
number of subunits in a.u.	12
Resolution (Å)	49.53 – 2.55<br>(2.64-2.55)
R_{sym} or R_{merge}	0.149 (0.859)
R_{pim}	0.079 (0.455)
$<I/\sigma(I)>$	7.7 (1.7)
Completeness (%)	99.2 (61.9)
Redundancy	5.3 (5.1)
**Refinement**	
No. reflections	64,409
R_{work} / R_{free}	0.1872 (0.2479)
No. atoms	
Protein	13728
Solvent / Fe ions	287 / 12
R.m.s. deviations	
Bond lengths (Å)	0.009
Bond angles (°)	1.08
Ramachandran plot (%)	
Favored	99.17
Allowed	0.83
Outliers	0
Rotamer outliers	2.55
Cβ deviations	0

Supplementary methods

Assessment of the presence of Gram-positive and Gram-negative contaminants in recombinant CAIP preparations

Stably transfected HEK 293 hTLR4/MD2/CD14 cells (InvivoGen) were seeded into 96-well plates at the density of 3×10^5 cells/ml. Cells were transfected with Firefly luciferase reporter constructs, pGL3.ELAM.tk, and Renilla luciferase reporter plasmid, pRLTK as published[2]. HEK.293 hTLR4/MD2/CD14 were exposed to 100 ng/ml *E. coli* LPS (LPS-EB ultrapure, InvivoGen) or to 20 µg/ml CAIP. Cells were stimulated for 4 h and 18 h. Similarly, stably transfected HEK293 hTLR2 cells (InvivoGen) were exposed to 20 µg/ml CAIP or to 1 µg/ml Pam3CSK4 (InvivoGen). Stimulation was carried out for 6 h and 18 h. NF-κB-dependent luciferase activity was measured at 4 h (for hTLR4/MD2/CD14) and 6 h (for hTLR2) using the Dual-Luciferase Reporter Assay System (Promega, Fitchburg, WV, USA), as reported[2]. IL-8 release was quantified at 18 h of stimulation by ELISA.

Evaluation of the impact of LPS contamination on the cytokine expression induced in monocytes by CAIP

2×10^6 monocytes were exposed to 20 µg/ml CAIP (boiled or not), to 100 ng/ml LPS (boiled or not) or saline (negative control) for 2 h. Total RNA was extracted from 2×10^6 monocytes using TRIzol solution (LifeTechnologies) and reverse transcribed using SuperScript II (LifeTechnologies). cDNA was amplified with the following primers: 5′-AGCAACAGGGTGGTGGAC-3′ and 5′-GTGTGGTGGGGGACTGAG-3′ for GAPDH; 5′-ACAAAGGAGGCGAGGTTCTAA-3′ and 5′-CCCTTGGGGGTCAGAAGAG-3′for IL-12p40; 5′-TCCACCAGGGTCTGATTTTT-3′ and 5′-TTGAAGCGGAGAAGGAGACG-3′ for IL-23p19; 5′-CTGTCCTGCGTGTTGAAAGA-3′ and 5′-TTGGGTAATTTTTGGGATCTACA-3′ for IL-1β; 5′-AACCTGAACCTTCCAAAGATGG-3′ and 5′-TCTGGCTTGTTCCTCACTACT-3’ for IL-6; 5’-ATGAGCACTGAAAGCATGATCC-3’ and

5’-GAGGGCTGATTAGAGAGAGGTC-3’ for TNF-α. After amplification, data analysis was performed using the second derivative method algorithm by applying the 2^−ΔΔCT method. For each sample, data were normalized to an endogenous reference gene (GAPDH). Negative control cells were taken as the reference value and the relative expression levels for treated cells were calculated and shown.

Crystallization, data collection and crystal structure determination

Crystallization screens were carried out at 20°C by vapor diffusion technique using an automated sitting-drop setup (Oryx-8, Douglas Instruments). Two crystal forms were obtained: cubic-shaped crystals that grow in few days and very small and tiny plates that need some months to grow. Whilst the first crystal form diffracts very poorly, the spectrum of the second extend to a maximum resolution of about 2.5 Å. The latter were grown starting from a solution containing 0.1 M SPG (pH 4.0) and 25% w/v poly (ethylene glycol) 1500 as a precipitant (solution number 1 PACT Premier HT-96 kit; Molecular Dimensions). Data were measured on the ID23-2 micro-focus beamline of the European Synchrotron Radiation Facility (ESRF, Grenoble, France). 2720 images with an oscillation range of 0.1° and an exposure time of 0.04 s were collected. Crystal are monoclinic, space group P21, and they contain an entire biological entity, corresponding to twelve polypeptide chains, in the asymmetric unit (VM= 2.63Å3Da-1, solvent content 57%).

All datasets were indexed and integrated with software XDS[3] and merged and scaled with Scala[4]. The structures was solved by molecular replacement using the software Phaser[5]. The model was displayed and manually adjusted with graphic software Coot[6]. Refinement was carried on using package Phenix[7]. The final structure presents a final R factor of 0.1872 (Rfree 0.2479). Geometrical parameters of the models are quite good, particularly for a structure at this resolution. Data collection and refinement statistics are summarized in Supplementary Table 1. Data deposition Atomic coordinates and structure factors have been deposited at The Protein Data Bank (PDB) for immediate release as 5LBH.

Characterization of the metal binding site of CAIP

CAIP dodecamer possesses four three-fold axes, each of them passing through the shell in two different 3-folds environments arranged as pores. One of the two three-fold type pores corresponds to the iron entry channel postulated for the other members of the family. The strongly hydrophilic and negatively charged character of this pore is fully conserved with respect to HP-NAP. On the contrary, the other pore type is smaller and less negatively charged.

A Fe ion was fitted in the site occupied by the iron in the other members of the family, for a total of 12 iron sites. These cations represent the ferroxidase centres, where Fe^{2+} from the environment is internalized and oxidized, before being stored in the inner cavity. The ion present in CAIP displays a distorted tetrahedral coordination, where three corners are occupied by protein atoms, two oxygens and one nitrogen. They derive from the side chains of Asp51, Glu55 from a subunit and His24 from a nearby one. The fourth coordination position is occupied by a single atom that we interpreted as a solvent molecule. Nevertheless, its mean distance to the Fe ion (around 2.6 Å- 2.8 Å) and the fact that some residual density is present in the Fourier-difference map suggest that a heavier atom is present in this position. Other Dps-like proteins contains a di-iron site, but in this case this putative second cation does not present any coordination, being only linked to the Fe ion.

Supplementary References

1. Amedei, A. *et al.* The neutrophil-activating protein of Helicobacter pylori promotes Th1 immune responses. *J. Clin. Invest.* **116,** 1092–1101 (2006).
2. Paciello, I. *et al.* Intracellular Shigella remodels its LPS to dampen the innate immune recognition and evade inflammasome activation. *Proc. Natl. Acad. Sci. U.S.A.* **110,** E4345–54 (2013).
3. Kabsch, W. Integration, scaling, space-group assignment and post-refinement. *Acta Crystallographica Section D Biological Crystallography* **66,** 133–144 (2010).
4. Evans, P. Scaling and assessment of data quality. *Acta Crystallographica Section D Biological Crystallography* **62,** 72–82 (2006).
5. McCoy, A. J. *et al.* Phasercrystallographic software. *Journal of Applied Crystallography* **40,** 658–674 (2007).
6. Emsley, P., Lohkamp, B., Scott, W. G. & Cowtan, K. Features and development of Coot. *Acta Crystallographica Section D Biological Crystallography* **66,** 486–501 (2010).
7. Adams, P. D. *et al.* PHENIX: a comprehensive Python-based system for macromolecular structure solution. *Acta Crystallographica Section D Biological Crystallography* **66,** 213–221 (2010).

a

```
CAIP        MSKVVELLKQIQADASVFYVKVHNFHWNVKGMDFHPTHKATQEIYEQFADVFDDVAERVL    60
HP-NAP      -MKTFEILKHLQADAIVLFMKVHNFHWNVKGTDFFNVHKATEEIYEGFADMFDDLAERIV    59
              *..*:**::**** *:::*********** **. .****:**** ***:***:***::

CAIP        QLGEMPYVTLADMLKAAKIKEESKTSFCSKEIAQAVLADYEYFLKLFTELSAQADSQGDK    120
HP-NAP      QLGHHPLVTLSEALKLTRVKEETKTSFHSKDIFKEILEDYKHLEKEFKELSNTAEKEGDK    119
            ***. * ***:: ** :::***:**** **:* : :* **::: * *.***  *:.:***

CAIP        VSAAYADDKVGELQKAIWMLKSQLA    145
HP-NAP      VTVTYADDQLAKLQKSIWMLQAHLA    144
            *:.:****::.:***:****:::**
```

b

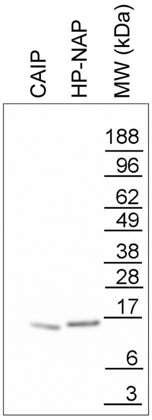

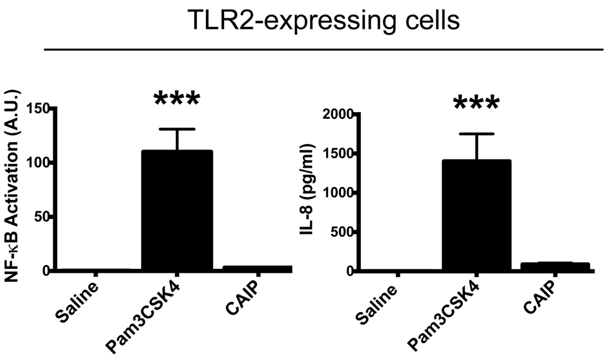

TLR2-expressing cells
NF-κB Activation (A.U.)
150
100
50
0
***
Saline
Pam3CSK4
CAIP
IL-8 (pg/ml)
2000
1500
1000
500
0
***
Saline
Pam3CSK4
CAIP

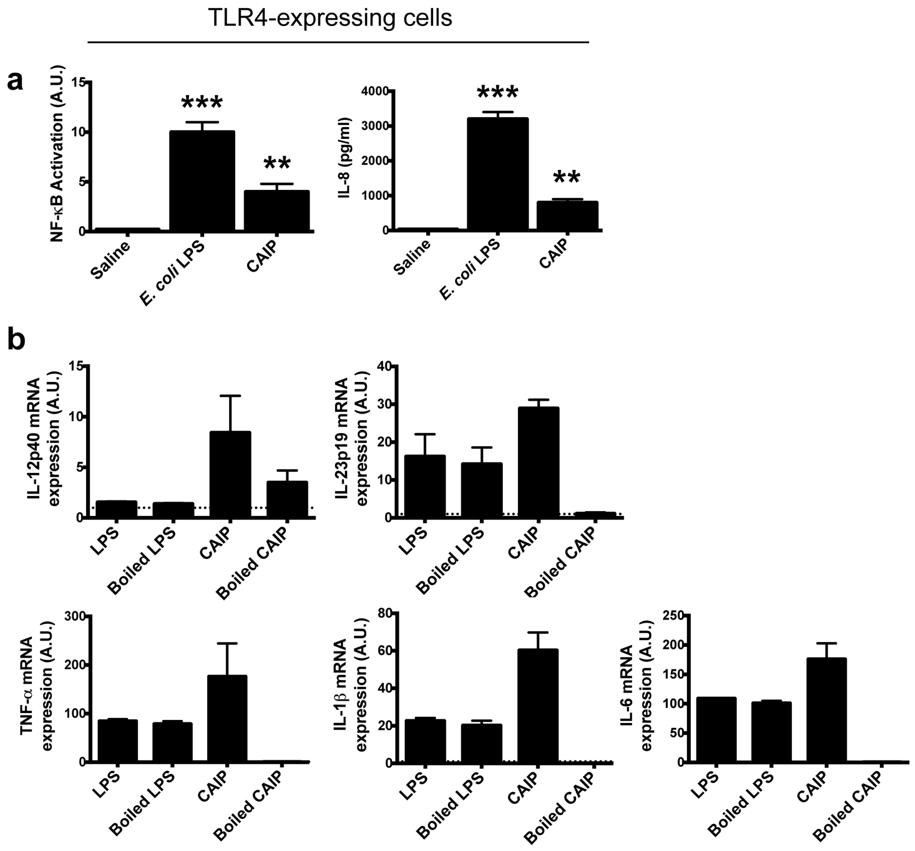
TLR4-expressing cells
a
NF-κB Activation (A.U.)
***
**
Saline
E. coli LPS
CAIP
IL-8 (pg/ml)
***
**
Saline
E. coli LPS
CAIP
b
IL-12p40 mRNA expression (A.U.)
IL-23p19 mRNA expression (A.U.)
TNF-α mRNA expression (A.U.)
IL-1β mRNA expression (A.U.)
IL-6 mRNA expression (A.U.)
LPS
Boiled LPS
CAIP
Boiled CAIP

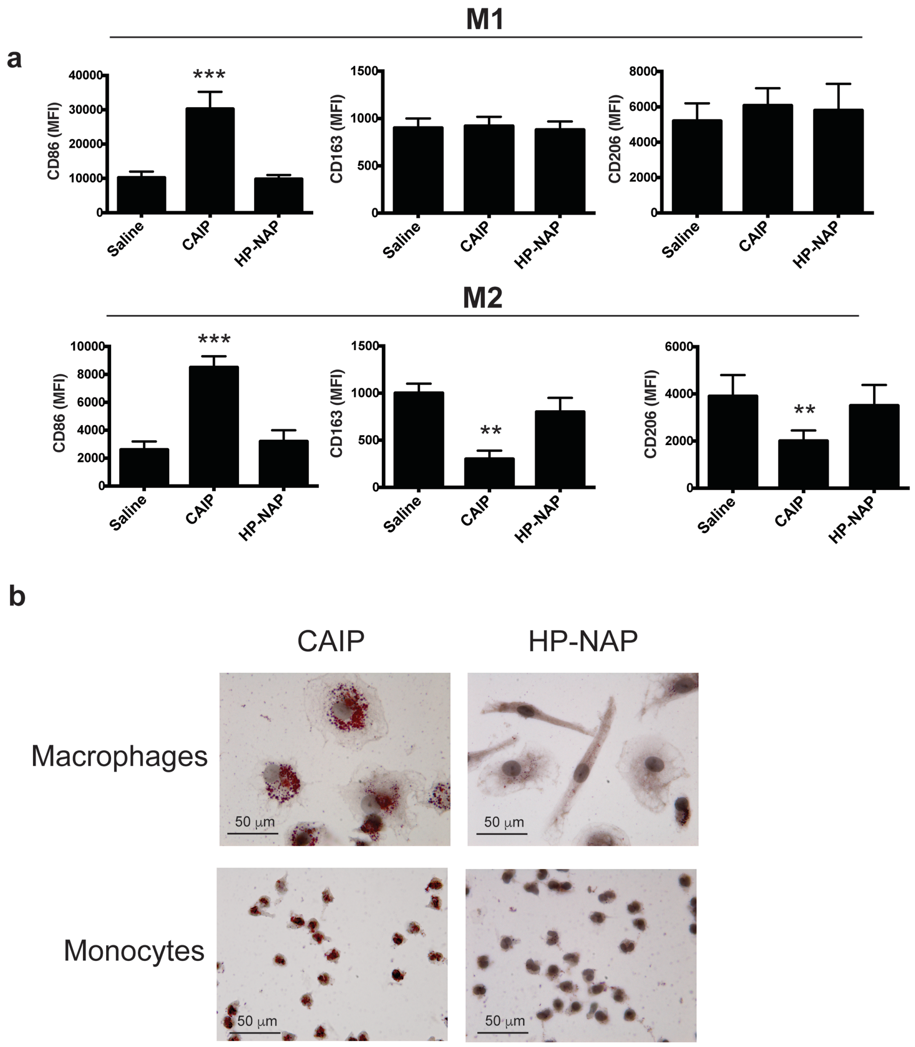
M1
a
CD86 (MFI)
CD163 (MFI)
CD206 (MFI)
***
Saline
CAIP
HP-NAP
M2
**
b
CAIP
HP-NAP
Macrophages
Monocytes
50 μm

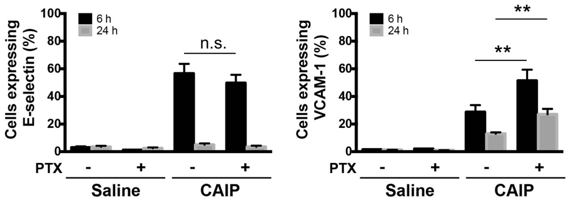

Cells expressing E-selectin (%)
100
80
60
40
20
0
6 h
24 h
n.s.
PTX
-
+
-
+
Saline
CAIP
Cells expressing VCAM-1 (%)
100
80
60
40
20
0
6 h
24 h
**
**
PTX
-
+
-
+
Saline
CAIP
